# Advancing Occupational Medicine through Wearable Technology: A Review of Sensor Systems for Biomechanical Risk Assessment and Work-Related Musculoskeletal Disorder Prevention

**DOI:** 10.1021/acssensors.5c01578

**Published:** 2025-07-17

**Authors:** Abubakar A. Babangida, Yohama Caraballo-Arias, Francesco Decataldo, Francesco Saverio Violante

**Affiliations:** † Occupational Medicine Unit, Department of Medical and Surgical Sciences, Alma Mater Studiorum University of Bologna, 40138 Bologna, Italy; ‡ Division of Occupational Medicine, IRCCS Azienda Ospedaliero-Universitaria di Bologna, 40138 Bologna, Italy

**Keywords:** wearable sensors, biomechanical risk assessment, work-related musculoskeletal disorders (WRMSDs), occupational health, inertial measurement units (IMUs), electromyography (EMG), ergonomics, personal protective equipment (PPE)

## Abstract

Work-related musculoskeletal disorders (WRMSDs) remain a major occupational health concern globally, and the conventional techniques for assessing them suffer some drawbacks. Indeed, conventional observational techniques are faced with subjectivity and the absence of real-time quantitative data; these emphasize the need for improved biomechanical risk assessment tools. Wearable sensor technology, which is considered an improved assessment tool, has received considerable acceptance in the occupational health field for evaluating biomechanical risk and preventing WRMSDs, focusing on their essential features and workplace significance. However, studies that have documented wearable sensors for biomechanical risk assessment focus mainly on the established sensing mechanisms, while the emerging wearable sensors are still in their infancy. This work aims to offer a comprehensive review of existing sensing mechanisms for biomechanical risk assessments, highlighting both established and emerging technologies for the advancement of wearable sensor systems that minimize ergonomic risks. Additionally, it serves as a guide for future research in wearable sensing technology for biomechanical risk evaluation. A comprehensive literature search was conducted across three databases, namely, Web of Science, PubMed, and Scopus; after the initial screening and removal of duplicates, 522 articles were identified, with 176 being included in the review. This Account discusses the working principles, applications, and limitations in occupational medicine, focusing on various types of wearable sensors, such as optoelectronics, soft wearable sensors, inertial sensors, pressure sensors, and electromyography (EMG) sensors. Moreover, this study offers an exhaustive classification of wearable sensors, emphasizing their development and incorporation into personal protective equipment (PPE). To improve ergonomic interventions and techniques for biomechanical risk assessment, this work promotes the incentive of quantifying ergonomic frameworks, real-time feedback systems, and standalone wearable devices. Our review also identifies key challenges, such as sensor placement, data processing, and worker acceptance, and proposes future directions for improving wearable sensor systems, including sensor fusion, miniaturization, and integration with PPE.

## Graphical abstract

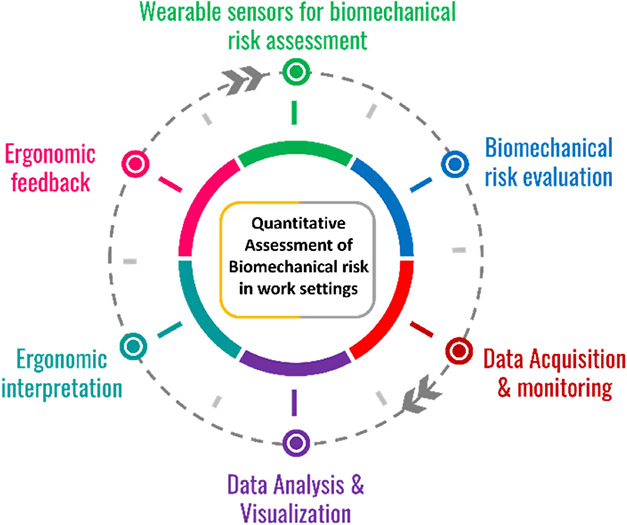

Wearable sensor technology has recently garnered attention in occupational medicine, primarily for its ability to bridge the gap between diagnosis and prevention of work-related musculoskeletal disorders (WRMSDs),^1–3^ which are highly prioritized as a public health concern in most countries. According to the World Health Organization (WHO) 2022 forecast, musculoskeletal disorders affect 1.71 billion people worldwide,^4^ leading to discomfort and functional impairment in various parts of the body. These disorders entail localized pain in areas of the body, which may be aggravated by exertion or external force,^5^ vibration,^6^ misalignment of affected body parts in motion,^7^ or prolonged static position/posture.^8^

WRMSDs are mostly caused by inadequately controlled occupational stressors that the body experiences while performing job tasks. These occupational stressors comprise both physical elements and biomechanical risk factors. Examples of these factors include manual material handling,^9^ prolonged or awkward work postures,^10^ excessive vibration,^11^ and fast, forceful upper limb movements with minimal or no breaks.^12,13^ The biomechanical risk factors are evaluated by identifying potential hazards in the workplace. The evaluation process begins with a thorough discussion of the worker’s job responsibilities, which requires a meticulous representation of all the procedures that are involved in a typical workday^14–16^ ; the degree of frequency, level of intensity, and the duration of each task in the work environment are considered in the analysis.

The predominant approach for assessing WRMSDs and biomechanical risk has been observational techniques. This is carried out through many methods, one of which is self-reporting via surveys administered in diverse formats. Experts utilize self-reporting across several contexts, such as interviews, documenting symptom histories or work-related activities, and completing individual questionnaires to articulate their symptoms. The other conventional observational approach involves manual visual assessment of the worker’s postural movements by the expert during work tasks, utilizing ergonomic assessment tools and note-taking for data collection.^8,17–19^ Although these techniques represent a standard method for WRMSD risk assessment, they suffer from important limitations. The results of the technique depend highly on the subjective opinion of the participants filling out the forms (i.e., being assessed), making it hard to draw a conclusion for the general population. These assessments do not yield valid and reliable estimates of either the exposure or the outcome. Also, the visual observation done by evaluators may be time-consuming, highly subjective to the observer’s perspective, which results in limited details and accuracy during gathering data.^20,21^

Visual-based tools, which include the use of cameras for 3D motion tracking, such as motion capture systems (Mocap), can be employed to improve accuracy, precision, and consistency of biomechanical risk assessments over the manual observational assessment.^22,23^ This evaluation is done by image capture, motion detection, or video monitoring of the kinetics and kinematics of workers as they perform their tasks. These visual-based tools involve the analysis of extensive data regarding workers’ overall activity, obtained from visual assessment devices. The monitoring procedure necessitates consistent calibration and is generally confined to structured laboratory environments. For instance, the observation of smaller body parts and rapid motion poses significant challenges, primarily because of the requirement for extensive calibration. Also, capturing the body kinematics is restricted to extensive use of advanced camera systems that are installed to operate within strictly controlled laboratory settings.^22,24^

The goal of developing exposure evaluation devices based on wearable sensors is to make WRMSD risk assessment more accurate and precise. In recent years, there has been a growing interest in the exploration of wearable devices in occupational medicine to ease current limitations and provide a feasible option for the continuous monitoring of personnel during work activities.^25–28^ Various wearable devices have been used for the analysis of biomechanical risk, the monitoring of posture and physiological parameters in occupational health.^29,30^ As previously noted, visual monitoring solutions generally confine observation to a controlled environment. Likewise, recently developed wearable sensors provide versatility and adaptability toward personalized work settings and environments, which is crucial for monitoring systems aimed at preventing WRMSDs, particularly in evaluating worker exposure and response assessment.^31–33^ In contrast to questionnaires, which are end-point reports of the working activities, wearable systems allow implementation of real-time feedback monitoring for prompt interpretation and evaluation.^34,35^

Implementing wearable systems in the workspace can provide enhanced quantitative analysis of individual workers’ health by generating highly reproducible and repeatable data, which can lead to the development of personalized smart protective equipment (PPE) (Figure 1), specifically designed to meet the workforce requirements.^30^ Compared to the previously mentioned assessment techniques, wearable sensors can decrease subjective biases and personal misinterpretations while enhancing personalized worker evaluation and customized protection. Despite the growing interest in wearable sensors for biomechanical risk assessment, most prior reviews have primarily focused on established sensor types such as inertial sensors and electromyography (EMG). Emerging sensor technologies are often either overlooked or only partially discussed with limited comprehensive analysis. Additionally, these reviews tend to concentrate on physically demanding work environments (e.g., construction), while neglecting other occupations with lower physical efforts that are nonetheless susceptible to work-related musculoskeletal disorders (WRMSDs).^36–39^ As a result, there remains a lack of comprehensive evaluations that address both established and emerging wearable sensor technologies tailored to occupational medicine across diverse industries. With this review, we aim to address that gap by providing a comprehensive study of all sensor types reported in the literature for evaluating and preventing biomechanical risk assessment, also emphasizing their essential features and significance in the workplace across various industries.

**Figure 1:**
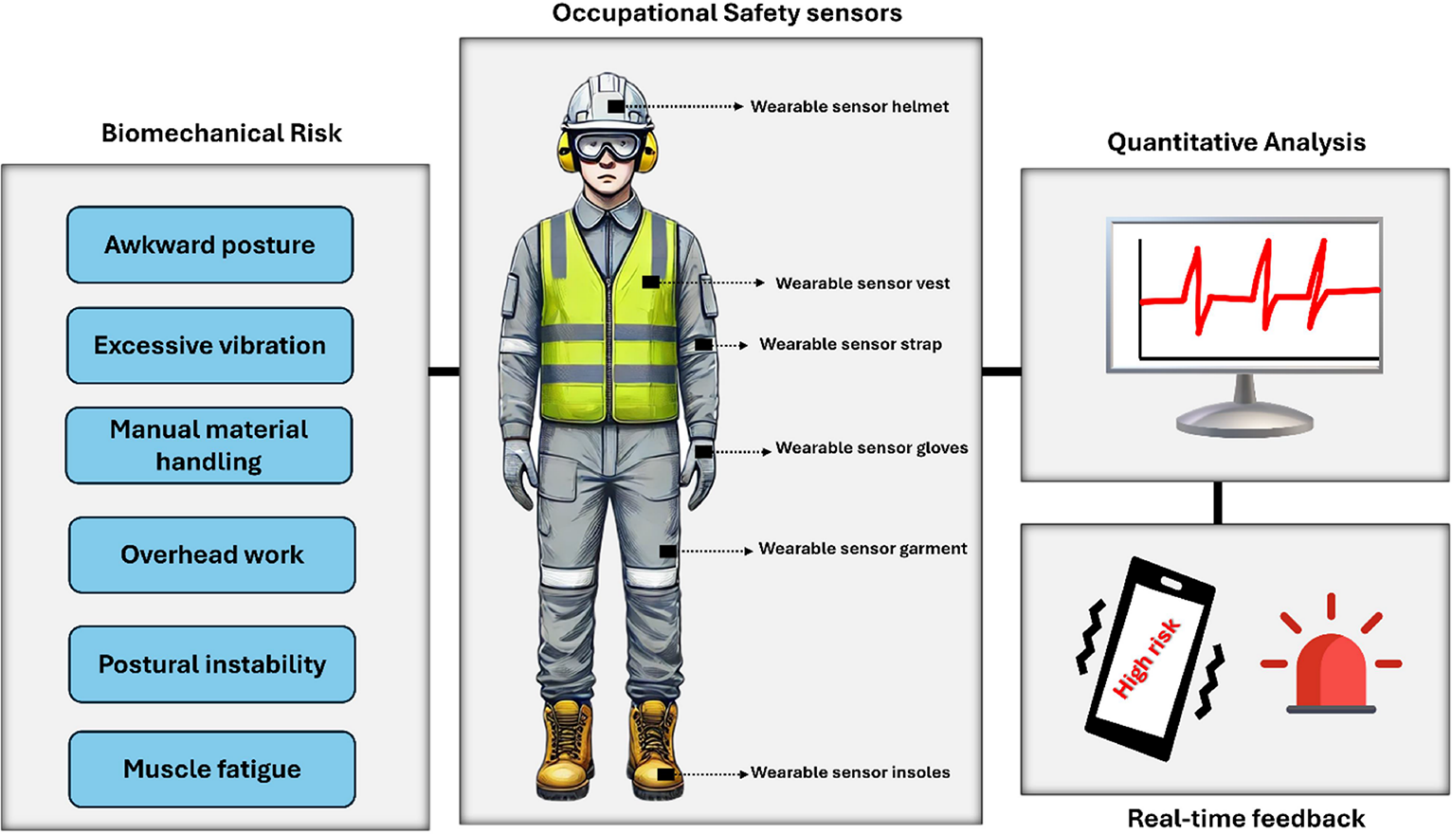
Quantitative Analysis of the Occupational Biomechanical Risk Assessment using wearable sensors.

This Perspective aims to provide a comprehensive evaluation of wearable sensors used for the quantitative assessment of biomechanical risks in occupational medicine. It explores both well-established and emerging sensing technologies, focusing on their working principles, body placements, and relevance to work-related musculoskeletal disorder (WRMSD) prevention. This work includes a comprehensive discussion of the integration of multiple sensors, their incorporation into personal protective equipment (PPE), and their compatibility with ergonomic assessment tools. Furthermore, this review examines critical factors influencing real-world usability, including user comfort, device interference during work tasks, and challenges associated with prolonged use. By also addressing the convergence of observational techniques and wearable sensing systems, this review highlights innovative strategies for enhancing risk detection and preventive interventions. We intend to address the following research objectives in our review:

- To identify and categorize wearable sensor types used in biomechanical risk assessment.
- To examine their sensing mechanisms, body placements, and applications in occupational medicine.
- To evaluate their integration with ergonomic tools and PPE.
- To discuss limitations and key challenges in current implementations, and
- To propose future research directions for improving wearable sensor systems in workplace ergonomics.

## Methodology

This section outlines the methodology used for the literature review, identifying and categorizing sensor types by detailing the eligibility criteria, search strategy, and screening process. These methodological decisions ensure that only studies relevant to biomechanical risk prevention in the work settings are included.

### Eligibility Criteria and Inclusion/Exclusion Parameters

The articles chosen for this literature search specifically focused on preventive measures related to biomechanical risk assessments in work activities. As a result, articles discussing sensors utilized for rehabilitation were omitted as they do not align with the focus on preventive measures. Articles lacking reports on the monitoring of biomechanical assessment procedures for work-related activities or experimental frameworks were considered to be ineligible for inclusion. Exoskeletons were excluded because they are more aligned with actuators than with sensors. We eliminated review articles as well as any books or chapters from the search to focus exclusively on peer-reviewed research articles. Therefore, the literature search consisted of all articles published in peer-reviewed journals or presented at conferences. The electronic search was carried out solely in English; as a result, articles not written in English were excluded to prevent any potential misinterpretation. Finally, we concentrated on examining and reading all of the peer-reviewed articles published from 2010 to provide an updated analysis of the state-of-the-art sensors within the scope of study. The authors examined the eligibility criteria, and in cases of disagreement regarding these criteria, the senior author rendered the final decision.

### Literature Search Strategy

A comprehensive electronic search was conducted in PubMed, Scopus, and the Web of Science databases to identify articles that have reported on wearable sensors for biomechanical risk assessment in occupational medicine (Figure 2). The terms utilized in the literature search were merged through logical Boolean operators or, when suitable, free text terms. The terms and their equivalents were incorporated into each database as follows: “wearable” AND (“sensors” OR “device” OR “electronic”) AND “work” AND (“musculoskeletal” AND (“disease” OR “disorder”) OR “biomechanical risk”). All results from each database were incorporated up to August 2024. Following the removal of duplicates, all articles that aligned with the scope underwent a thorough manual screening and evaluation based on their titles and abstracts. This study included a comprehensive assessment of all full-text articles for eligibility, with data collection carefully executed to reduce any bias.

**Figure 2:**
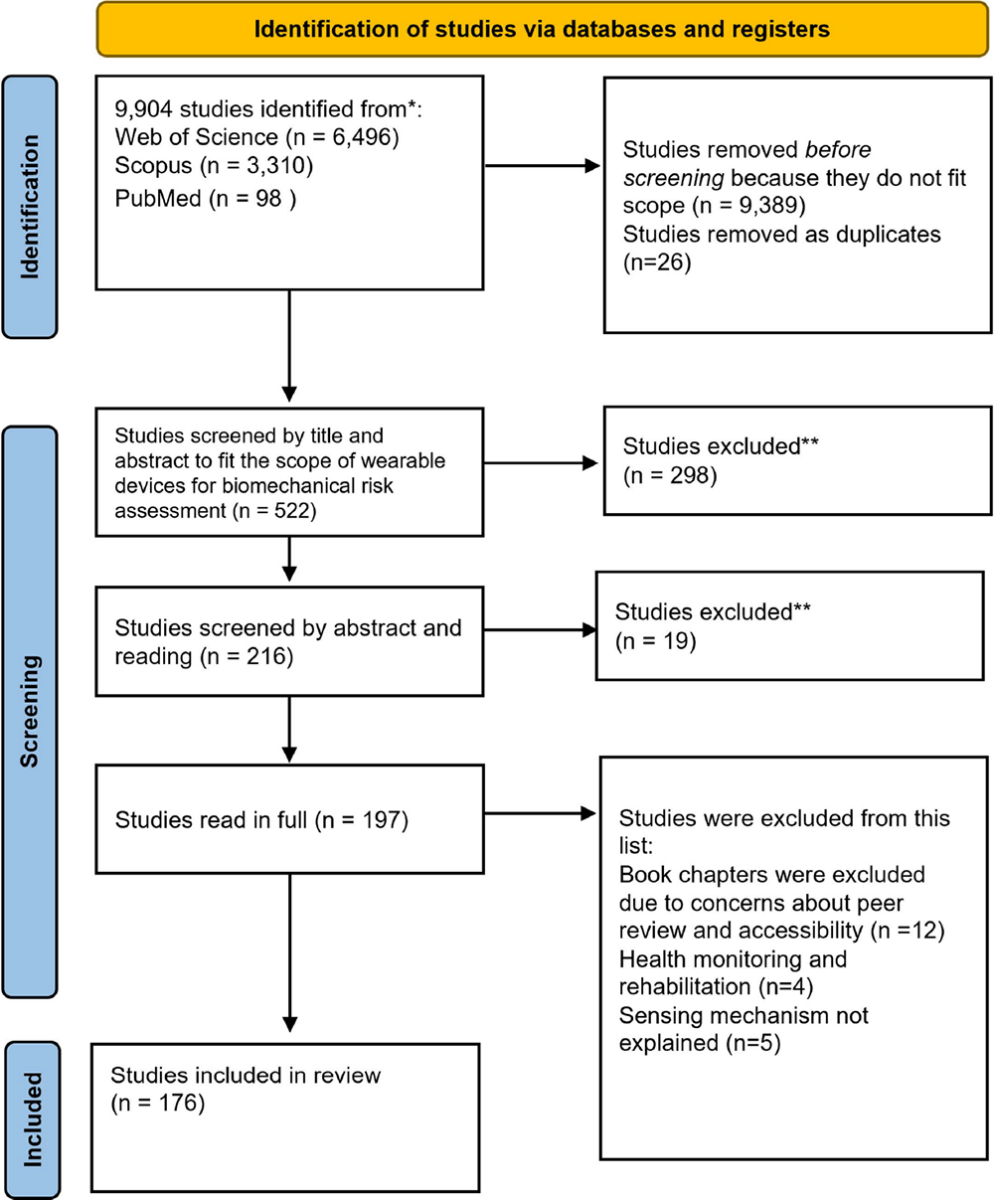
PRISMA flow diagram for the study selection process.

### Study Selection and Screening Process

A total of 9,904 records were identified via electronic searches across the three databases in August of 2024. Once the articles from the three databases were combined, duplicates were removed, and the remaining articles were screened by title and abstract for eligibility within the scope of our study. In the end, we identified 197 records suitable for full-text screening, leading to 176 studies being included in the review.

### Overview of Included Studies

All of the studies included utilized wearable sensors featuring various sensing mechanisms to conduct assessments of occupational medicine, with nearly all articles reporting on biomechanical risk assessment. Eight articles included monitoring of physiological parameters, while six articles focused solely on physiological parameters related to biomechanical risk assessment monitoring. Approximately 64% (*n* = 112) of the studies reported implementing at least one inertial sensor during risk assessment, highlighting the broad use of this sensing mechanism and the rationale for the implementation of these devices for biomechanical evaluation in work activities. The second most reported sensing mechanism was Electromyography (EMG), which was utilized in at least 32% of the total studies included (*n* = 55). The other notable sensing mechanisms reported in the included studies were the pressure insoles (*n* = 10), the optoelectronic sensor (*n* = 7), and the soft wearable sensors (*n* = 5), which included piezoresistive and piezocapacitive sensing mechanisms.

## Taxonomy of Wearable Sensors for Biomechanical Risk Assessment

Recent studies have shown significant interest in the development of various wearable sensors for biomechanical evaluations to mitigate occupational physical stressors that may adversely affect health and job performance. These electronics, based on various sensing principles, measure biomechanical factors: kinetics or kinematics behavior. Kinetic behavior involves load and force acting on a body during motion or stationary postures, whereas kinematic behavior is the analysis of motion, focusing on the whole body or in part without regard to the forces exerted during that motion. Thus, kinetic and kinematic factors in occupational biomechanics provide essential data for effective biomechanical risk assessment methods.^29,31–33,35,40–43^

This section outlines the various wearable sensors reported in the included studies based on their operational principles and the biomechanical exposures they measure. Also, it highlights occupations that may benefit from or can use these sensors in everyday work activities (Table 1), and a comprehensive comparison of the notable wearable sensors (both established and emerging devices) for biomechanical risk assessment in occupational medicine (Table 2).

**Table 1:** Overview of Wearable Sensors for Occupational Risk Assessment and Activity Monitoring

s/n	sensing principle	biomechanical risk	ref	body region monitored	sensitivity	range of detection	stability/repeatability	linearity	response rate/recovery rate/sampling rate	accuracy/mean accuracy error/error	activities monitored	occupations
1	fiber optic sensors	awkward posture	^46^	spine (lumbar region)	0.20 nm mε^–1^	increase of up to 2% of sensor’s initial length.	stable across 10 repeated cycles	*R*^2^ > 0.99	sampling rate = 100 Hz	∼16% or 0.33 cm	spinal range of motion in prolonged sitting	video terminal workers
2	fiber optic Sensors	awkward posture	^49^	spine (Thoracic region)	device 1,2,3 respectively: 32.32–44.87 pm/°, 23.89–38.29 pm/°, 35.13–50.45 pm/°	no information found	accuracy >96.3% for the 3 posture types	*R*^2^ = 0.944–0.999	real time monitoring (Response rate for Quiet breathing (QB): 0.07 nm and Tachypnea (T): 0.04 nm)	max RR error: 1 breath/min	sitting posture	video terminal workers
3	soft wearable sensors	manual material handling	^50^	hand	no information found	weight prediction between 1.1 to 17.0 kg	high stable results across 30 candidates tested	average peak gripping force with *R* ^2^ = 0.821	sampling rate = 25–40 Hz	no information found	lifting heavy weights of up to 20 kg	no information found
4	soft wearable sensors	awkward posture	^51^	spine (lumbar region)	no information found	no information found	no information found	*R*^2^ = 0.956	sampling rate = 100 Hz	mean angular error:	lumbar region posture detection	astronauts (intended use)
										flexion: 7.3° (11%)		
										lateral bending: 8.6° (15%)		
										rotation: 13.7° (28%)		
5	IMU and FBG sensors	repetitive loading	^40^	upper limb (Arm)	no information found	no information found	no information found	nonlinear due to sensor fusion	sample rate of 52 Hz for OF and IMU signals	accuracy of 98.4%	repetitive load task activities with increasing load	no information found
6	IMU and pressure sensors	awkward posture and manual material handling	^52^	spine (thoracic region) and the foot (insoles)	no information found	no information found	no information found	non linear model used for performance calculation	sampling rate of 100 Hz for both inertial and force sensors	minor error detected <6.5% of body height	manual lifting and assisting patients	health care workers
7	accelerometer	manual material handling and awkward posture	^17^	upper arm region	no information found	no information found	highly stable across 70 cycles of work shift	no information found	response rate = ∼100 Hz	no information found	manually handling baggage activities	flight baggage handlers
8	inclinometer	manual material handling	^53^	spine (trunk) and upper arm region	no information found	no information found	no information found	no information found	no information found	no information found	manually handling and sorting baggage	aircraft Baggage Handlers
9	accelerometer	awkward posture	^30^	spine (waist region)	no information found	no information found	no information found	linearity of *R* ^2^ ranging between 0.66 to 0.98	no information found	no information found	postural stability	rebar workers
10	gyroscope	manual material handling and awkward posture	^54^	spine (thoracic and lumbar region)	no information found	sensor able to detect ranges across 3-axis (all planes) of joints	no information found	no information found	no information found	accuracy between gyroscopes is 0.22 ± 0.09°	manual lifting activities	factory workers
11	EMG	repetitive manual material handling	^55^	spine	no information found	no information found	no information found	no information found	response rate = 2.56 s	accuracy of 98.3%	manual lifting activities	rebar workers
12	EMG	overhead work	^56^	upper limb (arm)	no information found	no information found	stability through milking cycle 60–90 min period on at least 50 cows	no information found	real-time capture, sampling rate 100 Hz	accuracy validated with observational S.I scores	milking the cows in upward posture	dairy workers
13	pressure sensors	manual material handling	^57^	foot (insoles)	high sensitivity >95.8%	no information found	consistency shown in >98% of repeated activities	no information found	real-time capture with sampling rate of 50 Hz	accuracy of 98.3%	manual lifting activities	construction workers
14	IMU	manual material handling and awkward posture	^58^	chest, spine and lower limbs	high sensitive up to 99.4% when detecting correct from incorrect posture	no information found	stability with repeated activities across 26 participants	no information found	real-time monitoring.	accuracy of 99.4% for full trunk + limb data.	manual lifting activities	construction workers
									sample rate = 40 Hz	accuracy ∼76.9% with trunk-only data set		
15	IMU	awkward posture	^25^	head and upper body	high detection rate of posture risk during surgical procedures	no information found	high stability across 47 participants	no information found	sampling rate = 128 Hz	sensor data validated using RULA scores	open and endovascular surgery procedure	surgeons
16	IMU and EMG	repetitive forceful exertion	^59^	spine, upper and lower limbs	high detection rate of posture and REBA scores	no information found	sensor stability in participants performing 4 task each	no information found	real-time data capture	strong correlations between EMG and pain (*r* > 0.94).	palm oil harvesting activities	palm oil harvesters
										REBA scores matched observational experimental data		

**Table 2:** Summarized Comparison of Notable Wearable Sensors for Biomechanical Risk Assessment in Occupational Medicine

sensor type	monitoring	biomechanical parameter	strengths	limitations	application reported	reported studies (ref)
IMU (accelerometer, gyroscope, magnetometer)	1-posture and movement	kinematics	1-high accuracy in motion tracking	1-sensitive to magnetic interference,	1-construction activities,	^25,30,58,91,124^
	2-velocity		2-real-time data	2-many units are needed for full-body tracking	2-repetitive lifting tasks	
	3-orientation		3-easy to wear			
EMG	1-muscle activity	kinetics (muscle force/activation)	1-detects muscle fatigue	1-bulky with multiple wires	1-manual Lifting jobs	^32,55,56,111,112^
	2-muscle fatigue		2- load classification	2-can be affected by sweat	2-monitoring awkward postures during surgery	
			3-good for strength-intensive jobs	3-requires calibration	3-overhead work	
pressure sensors	1-foot pressure	kinetics (force/load)	1-good for gait	1-limited to force/pressure	1-construction activities	^29,52,57,105^
	2-weight distribution		2-balance analysis	2- does not track motion	2-fall risk monitoring	
			3-integrates well with insoles			
optoelectronics (fiber optics)	1-spine and joint movement	kinematics	1-free from electromagnetic interference	1-lab-based mostly	1-video terminal activities	^44,46–48^
	2- posture		2-high flexibility	2-not widely commercial yet	2-sedentary tasks	
			3-integrated in wearable garments			
soft wearables (textile-based)	1-postural alignment	both (depending on integration)	ease of integration into PPE/clothing.	technology is at its early stages	1-video terminal activities	^50,60,64,65^
	2-strain				2-long sitting/standing postures	
	3-pressure					
inclinometers	angular body posture (e.g., spine, limbs)	kinematics	simple monitoring device	1-limited to one axis	1-baggage handlers	^53^
				2-not ideal for complex movements	2-warehouse work	

### Optoelectronics (Flexible Optical Fibers)

Optoelectronics use optical fiber strands as sensing elements that respond to variations in light intensity or wavelength to detect changes in strain, force, or other physiological parameters.^40–42^ One of the simplistic approaches for designing optoelectronics is a flexible fiber optics sensor, which is embedded as optical fiber strands within a silicone substrate. The sensor responds by emitting optical signals when the deformation encountered by the silicone substrate impacts the optical fiber strand: the light signal changes are correlated to the monitored physiological parameter. This principle has been used to design highly responsive wearables to assess biomechanical risks such as awkward posture: the evaluation involves applying the fiber optic sensor on the spine to monitor the range of spinal motion, with certain studies reporting assessments of sitting postures for video terminal activities during work shifts.^41,43,44^ Zaltieri and colleagues have worked closely with fiber optic sensors for ergonomic evaluation and reported some studies monitoring the spinal range of motion. In one of their studies, there was implementation of flexible sensors to detect the range of motion in the thoracic regions during prolonged sitting activities with good linearity (*R* ^2^ > 0.99), sensitivity of 0.10 nm mε^–1^. The biocompatibility and conformability of the device were noteworthy, as the sensors could be attached to any type of garment and could fit the curvatures of the body.^45^ Also, fiber optic strands were combined with silicone patches in a different work, designing smart e-textiles for the detection of awkward posture by monitoring dorsal movements in video terminal activities. The flexible sensor presented a highly linear response (*R* ^2^ > 0.99), sensitivity of 0.20 nm mε^–1^, while portraying good repeatability when observed through 10 mechanical trials and conforms to the natural curvature of the spine.^46^ In another study by Zaltieri et al., the flexible Fiber Bragg Grating (FBG) device monitors trunk movements for the detection of upright kyphotic, upright, and lordotic postures. The device could recognize the three postures with an average accuracy of up to 97.6%, compared to a referenced commercial device (Zephyr Bio Harness 3.0 (BH)).^47^

Optic fibers, being resistant to electromagnetic interference, are used to develop sensors for ergonomic evaluation devices that measure the lumbar range of motion or limb motion, as an initiative to substitute radiography, which poses risks associated with X-ray exposure.^40^ In addition, these devices are flexible, lightweight, and comfortable for prolonged wear, ensuring no discomfort while detecting awkward postures in video terminal workers (Figure 3).^48^ All of the studies mentioned above aim to prevent low back pain, investigating poor postures during video terminal activities, thus highlighting fiber optic devices as potential tools for the early detection of low back pain in the workplace.

**Figure 3:**
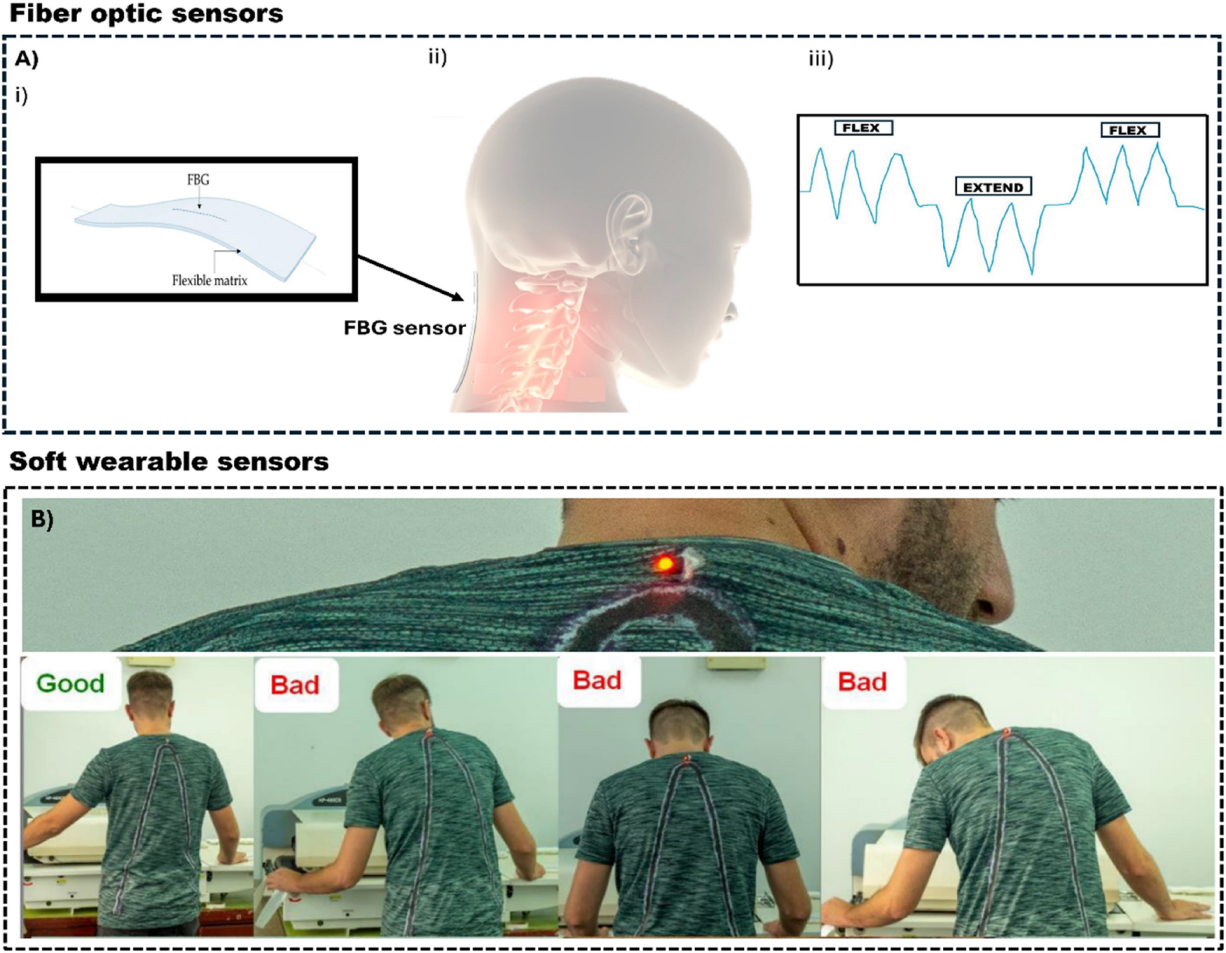
(A)­(i) Schematic illustration of a flexible fiber optic sensor and (ii) the fiber optic sensor placed on the neck. Reproduced from ref 48. (iii) Representation of signals obtained from FBG sensor during flexion and extension evaluation. Available under a CC-BY 4.0 license. Copyright 2020 Presti et al. (B)­(i) Textile-based piezo capacitive sensor for awkward posture detection and (ii) using the textile-based sensor to evaluate good and bad posture during work activity. Reproduced from ref 64. Available under a CC-BY 4.0 license. Copyright 2022 Maksimović et al.

**Figure 4:**
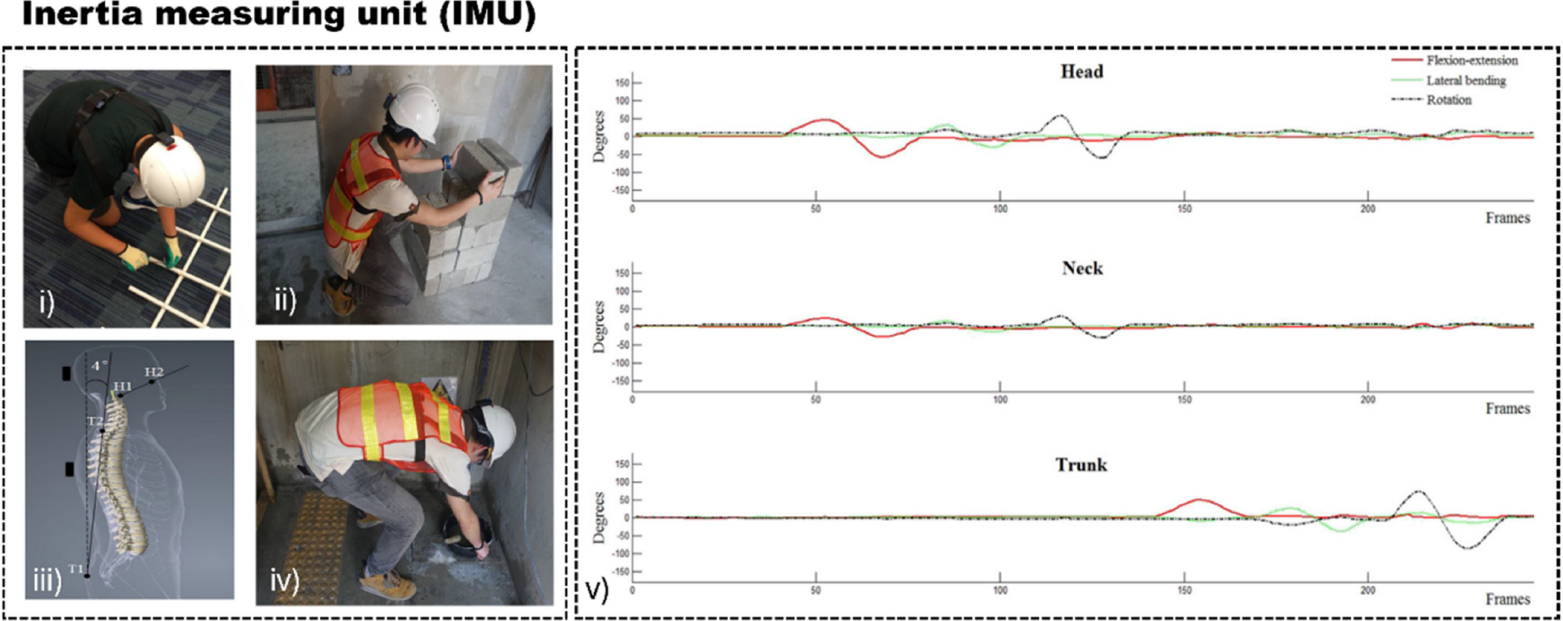
(i–iv) Position of the IMU sensor mounted on the spine of the worker and the range of spinal motion during job duties and (v) sensor detection of the head, neck, and trunk during biomechanical evaluation. Reproduced with permission from ref 100. Copyright 2017 Elsevier.

### Soft Wearable Sensors (Strain Sensors and Textile Sensors)

Soft wearable sensors for biomechanical assessment are a subject of significant interest as researchers want to create highly conformable devices appropriate for prolonged usage in professional environments and healthcare. The included studies that we analyzed involve soft wearable sensors, such as strain sensors and textile-based sensors. This is because researchers are leaning toward incorporating these soft wearable sensor electronics into either garments or personal protective equipment (PPE).^51,60,61^

Both strain sensors and textile-based sensors are designed using either resistive or capacitive working principles, reporting precise information on the shape deformation. Wearable sensors designed with resistive and capacitive properties have their response and sensitivity influenced by variations in external parameters such as pressure, force, stress, or strain.^62,63^ Maksimovic et al. employed a strain sensor composed of flexible capacitors made of electroconductive paint embedded in fabric to develop PPE aimed at monitoring body posture. This innovative design serves as standard working equipment intended to help rectify improper and high-risk upper body positions during extended and static work activities (Figure 3).^64^ In the work of Qian et al., the spine was monitored using a piezoresistive strain sensor to differentiate several sitting positions, ranging from normal posture to mild and severe hunchback postures.^500^ Similarly, a simplistic approach for fabricating e-textiles as protective gear was shown in the work of Patiño and colleagues: the smart e-textile was made by stitching copper wires onto elastic fabric. The preliminary analysis showed that the garment could facilitate the monitoring of the wearer’s back movements. However, because of the Young's modulus of copper wires, they might not be suitable sensing elements for the fabrication of soft e-textiles if comfort and stretchability must be considered.^65^

Piezoresistive sensors in gloves can identify biomechanical hazards associated with heavy lifting and repetitive tasks that can lead to carpal tunnel syndrome. In a study by Zhou and colleagues, a force sensing resistor was used to design a sensor that was integrated into a tactile glove to detect repetitive heavy object lifting. The gloves can detect weights up to 20 kg, with good linearity (*R* ^2^ = 0.821), and will trigger an alarm if the force sensing unit registers an unacceptable weight. Also, the gloves had a high reliability rate when tested among 30 participants and over 35,000 samples.^50^ Since gloves are recognized as necessary PPE for various occupations, including compact sensors into customizable workwear, it will provide workers comfort and flexibility without hindering their activity or introducing additional stress. Tessarolo and colleagues demonstrated a novel approach to creating e-textiles for protective gear. In their work, the sensor was fabricated by simply drop casting conductive polymer (PEDOT: PSS) directly on the textile substrate, designing conformable tactile gloves monitoring the hand during manual handling activities with the doped PEDOT: PSS having a sensitivity of 0.033 kPa^–1^ (<50 kPa) and 0.015 kPa^–1^ (>50 kPa) respectively.^60^ The straightforward fabrication method allows a low-cost and scalable protective wearable sensor in occupational health. Using the deposition method, composite materials are also a reliable method of fabricating simple soft wearable sensors on simple substrates like textiles and paper.^68^ A couple of these composite materials, such as nickel nano strands, nickel-coated carbon fibers, laser-induced graphene, and Mxene, have been used to fabricate soft wearable sensors for health monitoring. The application of these techniques to create cost-effective, flexible wearable devices for occupational purposes will enhance access to tools designed for monitoring biomechanical risks in workers.^66–68^

### Inertial Sensors

Inertial sensors constitute the most commonly used devices employed as wearable sensors for occupational health, thanks to their ability to integrate various sensing properties. The sensing elements of inertial sensors have been employed either independently or as a sensor fusion (i.e., combining data from multiple sensors) to create wearable sensors designed for the assessment of biomechanical risks. Wearable inertial sensors are important for the observation of human movements and the evaluation of balance. They monitor the kinematics of the wearer and subsequently provide the wearer’s new position, adding other meaningful parameters, such as velocity, orientation, gravitational forces, acceleration, etc.^3,54,69,70^ A combination of multiple sensing elements of inertia is termed as an inertia measuring unit.^71^ Wearable inertial sensors, such as inclinometers,^53^ gyroscopes,^54,72^ accelerometers,^73^ and inertial measurement units (IMUs),^20,74^ have attracted significant interest in biomechanics studies because of their cost-effectiveness and noninvasiveness while acquiring accurate kinematics data. Below, we will discuss some of the reported inertial sensors and IMU used for biomechanical evaluation of risk assessment related to WMRSDs.^75^

### Accelerometers

Wearable sensors made of accelerometers operate through the detection of motion and linear acceleration,^76^ evaluating several biomechanical factors. Ensuring optimal postural stability is essential for all workers, and the wearable accelerometer serves as a tool to assess postural stability across diverse working conditions, varying from high-risk to low-risk environments.^69,77^ A study by Guo et al. demonstrated that accelerometers are extremely effective for monitoring postural stability in masons during the laying of blocks and bricks on construction sites. The sensors placed on the body could have a maximum classification efficiency of up to 91.6 and 87.4%, respectively, and showed reliability across monitoring 30 participants and 24 postural activities. This study also highlighted that the pelvis and lower legs are recommended locations of the body for assessing the postural stability of construction workers, particularly the masons.^71^ Similarly, rebar workers, who are engaged in a high-risk occupation, can use accelerometer sensors to identify repetitive tasks and distinguish between various body movements during their job tasks.^30^ Also, healthcare workers involved in patient transfers may benefit from the accelerometer as an effective tool for evaluating their maneuvering position during work activities (forward bending and low back posture). This assessment could serve as a preventive strategy against WRMSDs, including low back pain and disorders affecting the upper limbs.^78^

Accelerometers can also track wrist and shoulder problems in occupations with physically demanding, repetitive tasks, such as those in slaughterhouses,^79^ in addition to measuring postural stability. In a particular study, the accelerometer was affixed to the participants’ wrists to monitor meat-cutting activities. The device achieved an impressive accuracy of 98%, allowing for a precise ergonomic evaluation of the risk levels faced by the workers,^80^ enabling the independent application of accelerometers for assessment and monitoring of postural risks for workers.

### Gyroscopes, Inclinometers, and Electro Goniometers

Wearable gyroscopes, inclinometers, and electro-goniometers can assess workers’ orientation as well as detect rotation of their body segments. Gyroscopes offer greater versatility compared to inclinometers and electro-goniometers: while gyroscopes detect movement across directions in all planes and angular velocities,^54^ inclinometers and electro-goniometers are only restricted to movement and bodily angles in a single plane, respectively.^53,81^ All of these sensors have been useful in the assessment of biomechanical assessment related to manual material handling activities, including upper limb elevation and angles. For instance, a wearable inclinometer device has been used to assess flight baggage handling activities, focusing on the trunk and upper limb postures to assist engineering or administrative initiatives in reducing exposure to postural risk that could lead to musculoskeletal disorders. The device could detect upper arm elevation as low as 10° and was highly reliable across 79 full shifts (651 h total) without noticeable signal drift.^53^ Lim and D’souza used gyroscope wearables to evaluate the manual handling of loaded goods, demonstrating their relevance for analyzing the trunk coordination of workers who routinely manage loads on both sides.^54^

### Inertia Measuring Unit (IMU)

The IMU incorporates a wide range of transducers for measuring motion and biomechanical movement relevant to occupational health, explaining its extensive use in the assessment of biomechanical factors related to musculoskeletal disorders. The IMU primarily comprises a magnetometer, accelerometer, and gyroscope, among other components, which are integrated by sensor fusion (i.e., combining data from multiple sensors) to operate as a single device.^82,83^

The use of wearable IMUs holds considerable potential for the practical application of some assessment tools (e.g., REBA, RULA), improving the understanding of these methods among medical practitioners who are accustomed to these methodologies. In addition, IMU can be useful to assist tools such as the NIOSH lifting equation in determining the recommended weightlifting posture associated with biomechanical risk.^84,85^ Incorporating the wearable IMU device with ergonomic tools enables more accuracy and reliability of these ergonomic risk assessments.^86,87^ Using wearable IMU with workplace-based exercise intervention to analyze and correct working posture is a viable alternative to physical ergonomics methods when they are easily accessible.^88,89^

The IMU device can be integrated into PPE to facilitate real-time analysis, which is advantageous for various professions, such as construction workers. This integration does not interfere with their operations and ensures gait stability in work environments, particularly on risky surfaces.^30,51,52^ Wearable IMUs exhibit considerable effectiveness in the evaluation of human motion during walking, particularly focusing on the lower or upper limbs.^86^ Studies indicate that the IMU can be employed to assess the risk of back injuries in workers engaged in load lifting, with the acquired signals validating biomechanical risk associated with this activity.^90^ In a study examining manual material handling activities that involve leaning forward, the magnetometer in the IMU proves effective in determining whether a user is bending their back or maintaining a straight posture.^91^ The IMU can identify and analyze the postures that workers adopt as comfortable for their work activities.^92^ It may identify postural positions that can aid in mitigating biomechanical risks associated with improper working postures during activities, such as manual lifting of heavy loads. These could lead to a better understanding of biomechanical factors and better training.^58^ In research using IMU for the assessment of gait stability, the device successfully recognized postural positions that aid in preventing spinal stress during awkward postures. The authors reported an impressive accuracy of up to 99.4% when employing appropriate kinematic parameters, and it was used to evaluate over 26 subjects while performing various repetitive tasks.^58^

It is essential to emphasize that the IMU devices depend on assessing the orientations of segments with a focus primarily on kinematics. These data are then employed to determine the locations of joints in the kinematic chain. However, it must be noted that load management plays a crucial role in evaluating musculoskeletal disorders in biomechanical activities (such as manual material handling), which involves analyzing the effects of different loads during these tasks. The presence of load in biomechanical risk analysis may lead to minor errors in measuring body segment positions. Difficulties in estimating joint locations and movements of the shoulders and hips can adversely affect the positional estimates of the lower limb segments.^92^ To enable IMU to thoroughly understand the kinematics of complex activities in manual lifting, several IMU sensors can be strategically positioned on various body parts to monitor the motion of the pelvis, wrists, and trunks of workers, facilitating the assessment of pushing and pulling forces.^93^

The IMU device can also be relevant for health workers, like surgeons, who often encounter increased tiredness and discomfort in the neck and lower back during or after open and laparoscopic procedures because of the awkward position. The IMU devices may aid in posture correction by tracking spinal curvature, neck angles, and upper limb movement.^20,94–96^ Similarly, the devices can be applied to nurses, who frequently maintain their trunk in a flexed position beyond the normal angle range while moving and assisting patients^97^; the data of the monitored and evaluated activity can be extracted and used to develop a solution for the investigated issue.^21,98^

Besides health workers, the IMU device can monitor office workers, who may be susceptible to neck and back pain because of improper posture; hence, the IMU can serve as a preventive instrument to observe spinal angle variations resulting from long sitting periods.^99^

### Pressure Sensors

Pressure sensors evaluate muscle kinematics and weight exertion in occupational activities and monitor gait stability and postural balance. In occupational safety, pressure sensors are used to thoroughly analyze the kinetics associated with biomechanical risks during work activities. The sensors are configured as dispersed pixels conforming to the surface of (or embedded into) workers’ PPE, targeting the precise area under investigation.^29,101^ They are mainly integrated into wearable insoles to evaluate fall risks associated with biomechanical risk assessments: the pressure matrices accurately detect the weight/pressure distribution of the wearer in hazardous environments, particularly in construction sites.^29,102^ This primarily addresses improper posture at construction sites during tasks that require maneuvering among workers (Figure 5) exposed to extrinsic fall risk factors.^103^ In addition, wearable insoles serve as a device to assess the risks associated with manual material handling, while simultaneously tracking the activities of workers, providing useful gait metrics to enhance the safety of the occupational environment.^104,105^ Antwi-Afari and Li employed pressure sensors integrated into an insole attached to a worker’s safety boots. The wearable insole sensing system included accelerometers to assess the kinematics of workers’ movements, while the pressure system on the insoles determined the kinetics of construction workers, as well as their gait stability and postural balance.^105^ The wearable insoles are versatile, enabling their use in various indoor and outdoor in-field monitoring settings, thereby significantly broadening their potential applications.^57^ It is noteworthy that these electronic devices can significantly improve current methods for recognizing loss of balance incidents and may assist safety managers in identifying hazards to apply proactive ergonomic techniques.^29^

**Figure 5:**
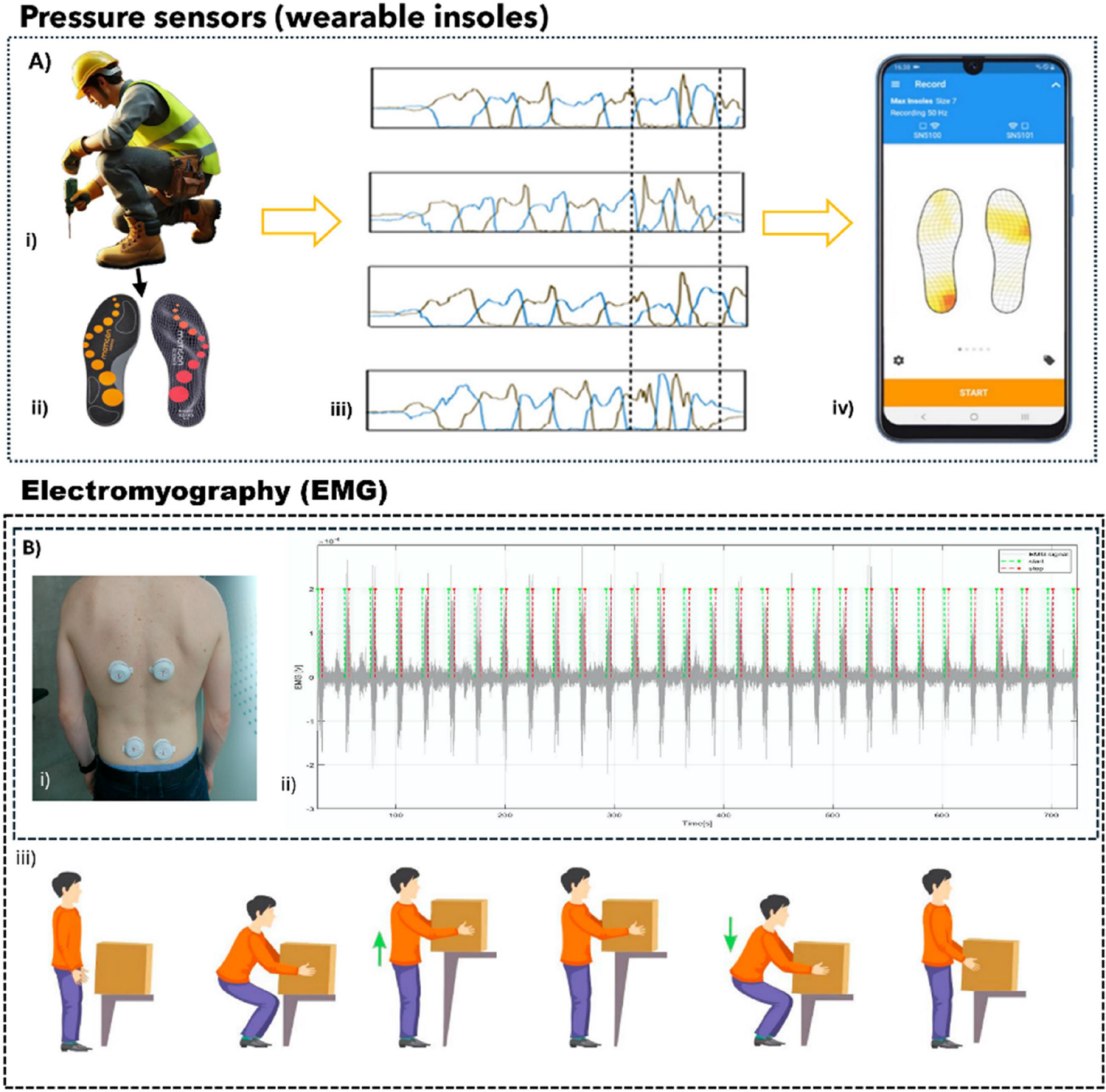
(i) and (ii) Worker’s ergonomic activity monitored using a wearable pressure sensor (insole). (iii) and (iv) Data output and topographical representation of force exerted during kinetic assessment. Reproduced with permission from ref 103. Copyright 2022 Elsevier. (B) (i) EMG configuration mounted on the erector spinae, (ii) EMG signals recorded from the pressure exertion on the muscle during kinetic analysis, and (iii) illustration of manual material handling activity evaluated. Reproduced from ref 32. Available under a CC-BY 4.0 license Copyright 2023 Donisi et al.

### Electromyography Sensors (EMG)

The excessive strain placed on muscles during strenuous manual handling tasks or repetitive tasks can lead to injuries, manifested as muscle fatigue, which subsequently increases the likelihood of developing WRMSDs in the affected body region. Wearable EMG sensors evaluate biomechanical risk by measuring and visualizing muscle activity in the monitored body segment, as an indicator of load intensity during ergonomic assessments, thus allowing for the classification of load levels into distinct categories, ranging from low to high fluctuations in force applied during tasks.^106–108^

Integrating EMG into wearable PPE facilitates the visual identification of body regions susceptible to overexertion.^109,110^ Varrecchia et al. used EMG sensors to monitor manual lifting activities and to determine which trunk muscles were most affected by changes in the lifting tasks. Their findings revealed that the erector spinae was the muscle group most responsive to weightlifting, thus offering a compelling rationale for managing the biomechanical risks associated with such lifting.^111^ In a similar manner, Taori et al. employed an EMG integrated into an armband to monitor lifting and lowering tasks, aiming to pinpoint repetitive manual lifting activities. The EMG wearable was identified as an appropriate ergonomic assessment tool, with a mean detection accuracy ranging from 79.2 to 86.9%.^112^

The EMG can also be used in situations concerning postural stability, awkward postures, and overhead work.^113–117^ Merbah et al. evaluated awkward postures using an EMG device. whose research focused on monitoring muscle fatigue during surgical procedures. The wearable EMG device monitored the musculature of the shoulder girdle and the cervical and lumbar regions of the spine, and the results suggest micro breaks play a beneficial role in mitigating muscle fatigue among surgeons during surgical procedures.^10^ Kim et al. conducted another study to assess muscle activity during green care farming activities, demonstrating the engagement of muscles in both the upper and lower limbs during various tasks, including farm clearing.^112^ Lastly, Masci et al. analyzed the relationship between repetitive tasks and awkward postures among dairy workers during milking, using the EMG sensor to evaluate the wrist flexor and extensor muscles.^56^

Sometimes, EMG devices can be bulky and may not be practical for use during specific biomechanical activities. This is because of the presence of numerous wires linked to the EMG during monitoring, which imposes a limitation that hinders usability and may lead to user discomfort. Thus, recent studies aim to address these constraints by developing wireless EMG devices with integrated battery powering systems, enhancing the practical application of these compact devices.^118–120^ Compact EMG wearables show a significant advantage in their ability to adapt more effectively to various working activities than ergonomic tools such as the NIOSH lifting equations and strain index (SI).^31,32,56^ For instance, the wearable EMG systems can be tailored to recognize lifting jobs pertinent to diverse professions (Figure 5), in contrast to the NIOSH, which has constraints in its applicability because of limited parameters and excludes many manual lifting activities from its criteria.^31,32,121^

Although the EMG clearly illustrates muscle activity and visualizes kinetics, it sometimes lacks the ability to monitor joint angles and body motion, which are valuable inputs for overall ergonomic evaluation. As a result, it is frequently used with kinematic tools like inertial sensors for location or motion detection. These kinematic sensors provide information on the range of motion for the executed activities, while EMG evaluates muscle stress.^107,122,123^ A study conducted by Antwi-Afari et al. that incorporated EMG and kinematic sensors reported that muscle activity during biomechanical tasks, such as manual lifting activities, does not significantly influence kinematics, regardless of the lifting posture used.^55^ However, integrating inertial sensors (kinematics) with EMG will enhance the overall precision of the wearable sensing system in biomechanical assessment evaluations.^118^

### Electroencephalography (EEG)

While the EEG cannot be used to directly assess biomechanical risks since it does not measure workers’ physical movements quantitatively, it can detect physiological data collected from gait and muscle movements. The application of EEG can facilitate the identification of workers’ behavior and cognitive performance during task execution, thereby evaluating operators’ mental fatigue at work. The use of EEG data to identify physiological parameters linked to biomechanical risk assessments could help reduce the likelihood of unsafe behaviors and hazards associated with biomechanical risks. This is relevant given that physically demanding tasks, like manual material lifting, impose a significant cognitive load during activity.^125,126^

Flexible headbands integrated with EEG technology can function as a tool for assessing mental fatigue at construction sites by analyzing patterns of brain activity. However, the application of EEG in real-world environments remains limited despite its potential to aid the evaluation of biomechanical risks. This is due to EEG devices requiring advanced algorithms and thorough noise filtering to ensure accurate data collection, which would limit this application to controlled laboratory settings.^126^ Although with recent developments, the EEG can be integrated with EMG for a thorough assessment of biomechanical risk factors in activities such as awkward postures and repetitive tasks. This research is in its preliminary phase, requiring further studies to validate these applications.^127^

### Heart Rate Sensors

Heart rate sensors can identify fatigue during work-related tasks; however, the relationship between heart rate monitoring and biomechanical risk assessment in occupational medicine remains ambiguous. Currently, heart rate sensors can track physiological data during intense activities, such as manual material handling or challenging repetitive tasks in construction workers, for the evaluation of physical fatigue. These sensors are mainly useful in demanding activities and fieldwork, to provide variations in physiological signals during activities and compared with data while at rest.^128–131^

However, it remains uncertain whether other biomechanical risk assessments can be evaluated outside of strenuous activities. Although combining a heart rate sensor with an EMG device or Inertia sensors appears to be an approach for integrating this device into biomechanical risk evaluation. A study showcased this application in the lower back during manual lifting, where the EMG tracked muscle activity in the lower back while the heart rate sensor distinguished heart rate activity throughout a loading curve. This method can assess the true risk linked to biomechanical factors and WRMSDs.^132,134^ A notable limitation of the heart rate sensors is the potential need for continuous monitoring of workers, both during their shifts and outside of work hours, to collect data for analysis.^43^

## Sensor Integration and System Design Approaches

This section highlights specific improvements that can be applied to wearable sensors, aimed at the optimization of these devices and the simplification of the assessment of biomechanical ergonomics by users, safety managers, and physicians. We discussed the sensor integration and proposed future improvements by exploring how different sensors can be combined and embedded into PPE. We also highlighted properties such as real-time feedback mechanisms, sensor fusion strategies, and smart workwear systems that optimize ergonomic interventions.

### Wearable Sensors with Active Feedback

Engineers integrate active feedback systems into or with wearable sensors as a form of proactive preventive mechanism, and researchers consider them potentially beneficial in enhancing the overall work ergonomics. Vibrotactile feedback is one of the common mechanisms used to improve the effectiveness of wearable sensing systems. They are actuators that provide necessary feedback through vibrations or sounds to ascertain the movement of workers, constantly evaluating their position to signal potential problems before they arise.^133–136^ The vibrotactile technology has recently gained popularity in the medical field as a therapeutic approach for Parkinson’s disease by incorporating it into gloves as a rehabilitation technique for reversing mild symptoms and acting as a preventive tool.^137^

These feedback devices, combined with wearable sensors, can be positioned on various segments of the body to notify the user when ergonomic thresholds are exceeded during physically strenuous tasks. The vibrotactile device for ergonomics operates by using a certain “rest” position/angle for the body segment, defining a range as the maximum limit of movement.^138^ If the worker exceeds this limit while performing a task, the device notifies an alert, sending vibrational signals, as a preventative strategy to avoid WRMSDs in that monitored body region.^85^ The vibrotactile device is appropriate for postural training in various professions. This device evaluates the working postures of the trunk and upper limbs, helping to decrease the duration of trunk inclination at certain angles associated with low back pain, thus reducing unsafe trunk postures.^134,139^ These devices can be combined with inertial sensors, as demonstrated by Kim et al., to evaluate the ergonomics of heavy lifting. The sensor fusion was placed on the spine and arms, assessing the lifting technique, with specific angles targeting the torso, arms, shoulders, and elbows, to identify biomechanical risks leading to accidents.^140^

The vibrotactile system is incorporated into the recent developments in ergonomics, which includes the enhancement of PPE into “Smart workwear systems”. These systems integrate inertial sensors into garments such as T-shirts, along with haptic feedback units, enabling the wireless transmission of recorded data of workers performing their duties.^141^ A simple implementation of these technologies into PPE was shown by Barone et al., where a wearable sensor integrated with a vibrotactile device was attached to a garment for measuring lumbar angles and assessing postural stability in sedentary workers.^142^ While there are differing opinions among workers regarding such wearable protective garments, there are favorable responses from those who find them comfortable to wear during work shifts. Zhang et al. conducted a survey involving 31 workers to assess their attitudes towards the use of sensors as part of PPE during work shifts; the participants were satisfied with wearing sensors while working.^143^ The “smart workwear system” (Figure 6) can help workers to adopt more comfortable and safer positions, providing feedback that can improve their work methods, workspace, and task design without a complete reshaping of the work environment.^134,141^

**Figure 6:**
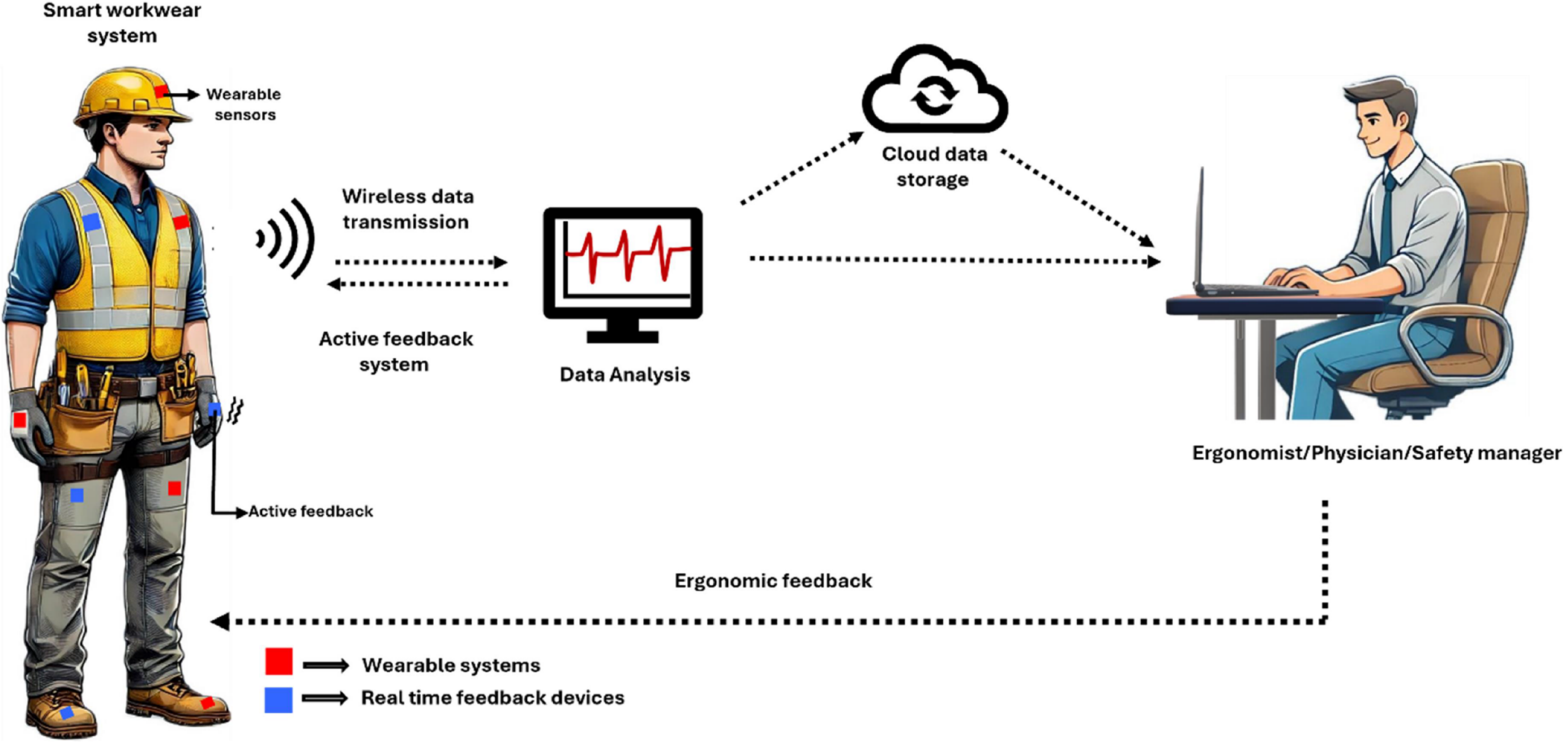
Smart work wear PPE was used to evaluate biomechanical assessment in work settings.

Other alternative feedback systems are real-time alert systems via software-based approaches that use personalized machine learning algorithms. These systems identify specific ergonomic tasks for evaluation and deliver immediate alerts through a connected display or application interface.^18,144,145^ Asadi et al. reported a Support Vector Machine (SVM) employed to distinguish between neutral and flexed postures in workers, achieving an accuracy of 96%. These models were then used to implement a system that would deliver real-time feedback via smartphones or industrial devices when thresholds were exceeded.^119^ Similarly, Giannini et al. implemented the Neural Network (NN) algorithm to identify manual material handling activities, issuing alerts when thresholds for awkward posture and ergonomic scores (from NIOSH and REBA) were surpassed during work activities.^146^ Also, Yan and colleagues used the alert feedback system to detect the posture thresholds defined by ergonomic standards (ISO 11226:2000), triggering an alert when the user exceeded those thresholds (Figure 4).^100^ Wu and colleagues have taken steps further in categorizing the alerting system according to individual evaluations: for instance, three short beeps showed incorrect joint angles; one long and two short beeps served as an alert for abnormal upper body posture; three long beeps signified prolonged sitting (>50 min) without movement.^99^ The alert feedback system is shown to serve as real-time feedback to workers for both improving the evaluation of biomechanical risks and preventing the development of work-related musculoskeletal disorders.

### Integration of Kinetics and Kinematics sensors

Sensor fusion involves the integration of data from various sensors, each operating on different principles, to enhance the monitored information. This process aims to provide a more reliable and accurate assessment of biomechanical risks, improving and expanding the overall data output of the wearable sensing system by utilizing the distinct characteristics of each sensor^52,147^ ; sensor combination leads to a highly responsive means that mitigates accuracy loss and optimizes individual performances.^40,148^ Sensor fusion is needed to prevent crosstalk and overlap during the monitoring of several joints. This is accomplished by integrating sensors with various sensing techniques or the capacity to identify biomechanical factors. Therefore, the selection of a sensor strategy is essential in this procedure.^149,150^ Van Gastel et al. integrated IMU and fiber optic sensors in the biomechanical motion tracking of container lashers. Each sensor’s unique characteristics complement one another, improving overall monitoring effectiveness. The fiber optic sensors show high linearity and resistance to magnetic interference, while the IMU demonstrated high accuracy and effective pinpointing properties.^151^

Van Gastel et al.’s sensor fusion only provides kinematic information on biomechanical evaluation; thus, we will report other sensor fusion that incorporates both kinematic and kinetic analysis below. It must be noted that evaluating biomechanical risk during activities involving lifting weights using only kinematic analysis has limitations. During manual material handling of loads, several factors beyond angular movements should be considered. Thus, a single wearable sensor for kinematic analysis may be only partially adequate to assess the biomechanical risk of several working activities in which musculoskeletal disorders are caused by a combination of kinetic and kinematic effects.^9^ Yoon et al. used a single IMU to evaluate manual lifting activities, and the monitoring process was supplemented with an optical monitoring system in a controlled laboratory setting to suit the optical capture.^152^ This is because biomechanical assessment using only kinematic sensors in the context of weight and load may require many distributed sensors across the body to evaluate the risk. Akhmad et al. used 9 IMUs and Kim et al. used 17 IMU devices mounted across the body regions to evaluate lifting activities.^84,153^ However, implementing kinetic sensors with kinematics sensors as a sensor fusion would effectively mitigate this issue, thereby facilitating a reduction in the number of sensors worn while simultaneously improving monitoring accuracy.^124,154^ For instance, a wearable device can implement IMU sensors on body segments such as the trunk, forearms, and areas of the spine for monitoring bodily movements; coupled with pressure insoles to provide an accurate representation of pressure mapping on the feet during strenuous tasks. This sensor fusion will allow complete biomechanical assessment with or without weight involved during the work shift.^52,155,156^ The final aim should be sensor integration into a complete PPE for industrial settings capable of evaluating both kinetics and kinematics data during working activities.

Also, sensor fusion can be realized with smart design, facilitating ergonomic assessment: i.e., not all of the sensors need to be worn by the worker. For example, Lee et al. integrated pressure sensors into office chairs with proximity sensors to assess risks linked to awkward postures and provide corrective measures. The pressure sensors were systematically positioned at the base of the chair, while ultrasonic sensors were mounted on the user’s back and monitored the distance from the backrest to the chair, enabling the assessment of whether the individual was seated in an ergonomic manner.^157^

### Combining Kinematics Sensors with Biopotential Sensors

The combination of IMU and EMG is considered an effective approach for the thorough evaluation of work-related musculoskeletal disorders. This facilitates the precise and continuous monitoring of limb movements and muscle activation levels simultaneously, resulting in the development of musculoskeletal risk assessments that surpass the accuracy of traditional ergonomic evaluation tools. Moreover, these devices (IMU- EMG sensor fusion) show better results for monitoring muscle fatigue when compared to single biopotential sensors.^158,159^ The multimodal sensors (sensor fusion) introduce innovative and streamlined methodologies for risk assessment. For instance, some researchers have digitized the Rapid Upper Limb Assessment (RULA) tool through integrating sensor fusion that includes the IMU and the EMG.^160,161^ Tahir et al. conducted research on the Digital Upper Limb Assessment (DULA) system, and the authors designed wireless wearable sensor intended for the digital computation of RULA scores. The data obtained from the wearable device can be validated through comparison with an online RULA calculator.^160^ The DULA wearable system shows high potential to be integrated into PPE for physical workers engaged in manual lifting and overhead tasks in various work environments. This approach overcomes the constraints of traditional monitoring using wearable sensors connected to a mobile application, enabling real-time risk assessment. Other forms of sensor combination, including kinematics and biopotential monitoring, have been used to calculate and predict the strain index,^162^ to evaluate manual material handling activities,^163–165^ and to analyze awkward postures.^59^ A couple of these integrated wearable devices have been tested in real work-scenarios such as for the evaluation of challenging outdoor duties during lashing containers,^146^ of workers tightening screws, and of other activities in an automotive assembly line production.^147,166^

This multimodal sensor system (sensor fusion) helps design an effective work environment by determining the number of hours required for a shift break, allowing the staff to recover from the stress of work in strenuous jobs.^167^ More studies have shown that the combination of kinematics and biopotential sensors can help to eliminate work stress, structure work shifts, and reduce the paperwork required by questionnaires.^147^ Beltran Martinez et al. used sensor fusion to investigate the effects of work shifts, both with and without breaks, during repetitive manual handling activities. The wearable system (IMU and EMG sensor integration) substituted workers’ questionnaire feedback for organizing work schedules: they demonstrated that workers’ fatigue levels decreased when they received necessary breaks after a work shift.^168^ An integrated sensor system monitoring kinematics and biopotentials would help to structure more effective work training, examining both expert and novice workers, studying how individuals adapt to work-related stresses, and evaluating the learning pattern of beginners as they progress through daily activities (for example, in highly repetitive heavy or fast weightlifting).^169^

With the sensor selection strategy, sensor fusion can be utilized to prevent crosstalk or data overlap, ensuring that monitored data does not result in cumulative interference. These sensors are arranged to avoid any overlap during activities and minimize cross-sensitivity from adjacent joints. This can be seen in the work of Tahir et al., where the DULA device combines IMU and EMG, with the IMU placed on the forearm to monitor movement and orientation, while the EMG is placed on the biceps to evaluate muscular activity. The processing unit of the device controls the synchronization of the sensors, ensuring the alignment of data across a coordinated time frame. This facilitates the simultaneous yet distinct detection of motion and muscle activation. In addition, it is essential for the sensors to achieve time synchronization to align with one another and to present results relevant to the same job duties. Also, interference between sensors caused by noise from connecting wires can be mitigated by reducing the number of wiring devices. This can be done by incorporating standalone wearable sensing devices with wireless data transmission and self-powering capabilities to decrease crosstalk during monitored work activities.^9,151,160,168^

## Critical Insights, Practical Challenges, and Future Outlook

This section addresses current limitations and outlines potential advancements in wearable sensor technologies. We identified some key challenges in usability, accuracy, and acceptance and highlighted prospects to enhance real-world applicability in occupational medicine.

### Limitation of Wearable Sensors

Despite wearable sensors having shown promising outcomes in biomechanical risk assessment, there are numerous constraints that must be addressed to provide effective monitoring in the workplace. Each sensor possesses unique characteristics; however, many of them show common limitations that require cautious consideration.

Specific issues related to kinematic sensors have been identified that need to be addressed to prevent challenges in kinematic analysis. For example, the magnetometer is particularly sensitive to magnetic interference, which can impact the assessment of certain activities when using IMUs. This renders IMU applications less suitable for environments affected by magnetic fields as working places having numerous motors, iron bars, and electrical power lines.^2,100,141,170^ Also, fiber optic sensors, electro-goniometers, and inclinometers are limited to measuring motion in a single direction. This limitation can hinder the worker’s ability to move freely in a practical work environment, thus failing in monitoring tasks that necessitate diagonal and combined movement.^46,47,147^

Certain job tasks that involve intense repetitive activities or maneuvering techniques could cause mechanical strain, such as bending, stretching, or twisting on wearable devices when sensors are integrated into PPE or placed directly on the skin. This mechanical strain might cause performance issues and make the devices more prone to calibration drifts, motion artifacts, and sensor weariness over time. This is common in flexible devices, since they are prone to mechanical deformation because of their heavy reliance on the substrate material for longevity and durability. Also, the IMU, EMG electrodes may experience motion artifacts or displacement during intense activities when placed on the PPE. This could result in noise signals and accuracy issues.^94,114,163,171^ In specific situations, there is a need to attach the sensors to the worker’s skin or to introduce additional straps and belts to fasten them firmly to the body segment during the evaluation processes.^164^ However, when sensors are attached to the skin, devices like the EMG may face constraints because of biological factors such as perspiration, which generates noise during data acquisition. This represents a major drawback for prolonged applications during manual material handling activities in industrial settings.^168,172^

Another critical issue is the small worker sample size monitored during the experiments, as they do not accurately reflect the larger population. To ensure broader applicability, it is essential to validate these devices by monitoring a larger and more diverse population, across various real-world working conditions and industrial settings to ensure validation and practical relevance.^100,114,119,173^ Many studies emphasize the significance of employing wearable devices for the evaluation of biomechanical risks over prolonged periods, rather than concentrating solely on short-term monitoring in workplace settings.^164^ Short-term studies cannot provide substantial insights into work-related musculoskeletal disorders. Likewise, data obtained from extensive work-related studies present a more precise representation of practical usage when compared to short-term studies.^56,80,122,174^

The results obtained from the studies may be affected by using specific wearables in controlled laboratory settings, limiting their predictive value and wider practicality.^10,31,48,175,176^ This shows that additional research is required to examine the dynamics of kinetic and motion factors within the workplace environment. For example, wearable devices may produce varying outcomes for participants free from low back pain (healthy workers) and those suffering from chronic lower back pain, in a systematic experimental framework of lifting activities.^123^

Conducting on-site assessments with these wearable systems is crucial for quantitatively evaluating efficiency in risk assessment analysis. Furthermore, standardized data processing and machine learning are critical for clean and reliable results. For example, certain sensor algorithms are only intended to analyze specific bits of raw data, not the complete monitoring process, and therefore are unable to effectively study the whole activity.^80^ Another constraint is that workers may lack confidence in using wearable systems because of the perception of being under constant surveillance during work shifts. Therefore, this impression must be addressed appropriately to encourage workers to adapt to the use of these devices.^171^

Lastly, commercial sensors still encounter some drawbacks that hinder their widespread use, which might need to be addressed. The use of too many sensors across the body impedes an accurate and comprehensive analysis of kinematics. This might discourage workers from wearing them during work shifts because of discomfort or distractions. In several studies utilizing the Xsens (MVN Link system), a total of 17 IMUs and 11 IMUs were mounted on different body segments to record body motion during manual lifting activities.^177–179^ This also applies to all commercial wearable sensors that require bulky wires to operate. Although most commercial sensors that depend on bulky wires (commercial EMG sensors and pressure insoles) have started the implementation of wireless data transmission through Bluetooth platform and connection, allowing easier and faster data collection.^52,162,180^ Thus, it is desirable to investigate the correlation between the discomfort of wearable systems associated with prolonged device usage over work shifts and their effect on accuracy levels. This will prevent the compromise of one in favor of the other.

### Commercialized Wearable Sensors

Several commercial wearables have been used consistently to study and improve wearable systems for the evaluation of biomechanical risks. The commercialization and accessibility of these electronics are crucial for realizing a significant impact through daily usage, as well as validation and integration of observational and questionnaire methods into quantitative assessments. Among commercialized wearables, the IMU stands out with an impressive commercialization rate thanks to its remarkable properties and ability to analyze kinematics: real-time risk assessment and feedback, wireless data transmission, a compact and wearable design, and minimal setup requirements.^181^ The commercial IMUs have been effectively employed in various studies to enhance ergonomic assessment methods; for instance, the Xsens system has been utilized by researchers to digitize the calculations of the NIOSH index and to assess RULA/REBA scores.^2,182^ After the IMU, the following most commercially available devices are the EMG and pressure sensors (gloves and insoles), which can conduct thorough kinetic analysis, distinguishing between various loads during both moderate and strenuous activities.^155,162,179,183^

Integrating sensors (commercial and custom laboratory-made ones) has enhanced the quality and quantity of research information that is important for occupational health and medicine. Commercial wearables are being designed with expertise to be versatile, allowing them to be seamlessly integrated into garments. Recent research has focused on making them more compact, ensuring that they fit comfortably on PPE without compromising accuracy. The commercial sensors are innovatively crafted as complete PPE solutions, including suits and insoles, equipped with haptic feedback to enhance the evaluation of work activities.^85^ A further advantage of commercial sensors within the research community is their utility as a benchmark for innovative wearable devices that are not yet available on the market. Current commercial sensors are establishing a standard for prospective innovations, as several of these sensors have been employed in experimental research within actual working environments.^147,155,184–186^ This will help in validating new wearable systems with improved performances for better evaluation of biomechanical risks.^40,187^

Lastly, commercial sensors can enhance the performance and functionalities of assistive “exosuits”, facilitating the design specifications tailored to specific occupations. This approach will help to identify optimal positions for integrating support points and incorporating joints in “exosuits”, and to address potential discomfort experienced by the wearer during activities involving combined movements (such as asymmetrical lifting tasks).^188^

## Prospectives and Outlook

Researchers are highly motivated to enhance and commercialize wearable sensors for biomechanical risk assessment in workplace applications. Their studies offer insights and approaches that advance current studies focused on optimizing wearable systems for consistent application. In this section, we highlight some of the valuable contributions considered relevant to the prospects of these wearable systems.

### Optimizing Sensor Placement and Device Ergonomics

Some studies have proposed that (i) proper placement of wearable sensors on the worker’s body and (ii) the device alignment/match with the specific work activities to be monitored are essential. This entails the careful design of devices to meet the professional standards of the human body or enhance their configuration to effectively reduce motion artifacts and noise signals during assessments. For instance, the IMU may be placed on the trunk or thighs by using sensor strap belts related to risk and fall detection. In contrast, for professions that employ tool belts, the IMU may be placed at the upper limbs with strap belts to effectively secure devices on the body during overhead work activities.^90,124^

The design of these wearables should integrate an intelligent workwear solution, characterized by a lightweight and adaptable system that can be seamlessly attached to garments, or PPE, or a wearable wrist armband (i.e., for tasks that engage the arms).^47,48,80,151^ This could involve looking into possibilities such as alternative design strategies or materials choices to ensure that these devices are durable and comfortable over typical work shifts and environments.^64^ For instance, the IMU, primarily designed in a rigid configuration, can be replaced with flexible substrates to fabricate the soft IMU, to facilitate the ease of integrating these devices into flexible PPE or skin for biomechanical risk assessment. The soft IMU has demonstrated considerable potential in health monitoring applications, such as tracking the early development of infant movement.^189^ Similarly, soft mechanoacoustic sensors with IMU, designed to be flexible and conformable, could be a promising device for enhancing the monitoring of worker behavior and ergonomics. The acoustic sensing mechanism combined with the IMU can facilitate the assessment of biomechanical and physiological parameters, all within PPE for effective monitoring.^190^

### Advancing Device Miniaturization and Cost-Effectiveness

Attempts can be made toward strategies that involve miniaturizing wearable electronics and integrating them with sophisticated features, such as wireless systems, for efficient real-time assessment.^47^ Also, it is important to make these wearables as cost-effective as possible to enhance their accessibility for ergonomic assessments, especially in highly structured facilities with limited resources.^113,114^ This involves utilizing cost-effective approaches using scalable manufacturing techniques for flexible sensors and a greater adoption of alternative low-cost materials for sensor fabrication to increase the wide adoption of wearable devices^60,66,67^ And most importantly, designing sensors with standalone sensing properties, to help minimize the use of wiring devices and enable wireless data transmission with self-powering capabilities. This minimizes any interference resulting from connecting wires during ergonomic evaluation.^191^

### Enhancing Multimodal Sensing Systems

Further improvement in multimodal sensing to integrate kinematic analysis (postural stability) with kinetic evaluation (weight distribution) would enhance the understanding of the relationship between extreme unsafe postures, manual handling activities, and WRMSDs. For example, combining sensors like IMU and pressure insoles would enable complete evaluation of activities associated with awkward postures and heavy lifting in risky activities in various industries.^53,192,193^ In addition, it is essential to validate and compare the results of emerging wearable electronics in the laboratory with commercial wearable devices, to accurately identify biomechanical parameters that differentiate healthy individuals from those with WRMSDs.^123,194^

### Improving Validation in Real-World Industrial Settings

There is a necessity for further experimental studies including larger population sample sizes and diverse, real working conditions in industrial settings to validate system performance,^29,147^ ensuring that low-cost devices are reliable for risk assessment in field evaluations.^113,176^ It is necessary to optimize and simplify the monitoring process by employing innovative strategies for wireless data transmission and enabling real-time feedback via mobile applications. These should be interactive and user-friendly for the improvement of occupational safety initiatives.^29^ More ergonomic evaluations can be conducted in an experimental study to give a better visualization of biomechanical risk assessment. For instance, evaluation of manual material handling using wearable sensors should not be limited to fundamentals such as picking up and dropping off objects; the experimental framework should include all activities that can lead to musculoskeletal disorders (e.g., pushing and pulling objects, or upper body twisting).^195^

### Streamlining Data Interpretation with Machine Learning

Machine learning algorithms, such as DULA, REBA score, and *k*-score (kinematic score), are essential for enhancing classification accuracy and minimizing noise in activity detection. These algorithms should cover the entire monitoring process, making it easy for safety managers and health professionals to easily interpret and comprehend biomechanical risk prediction and evaluation.^30,47,105,160,161,196–198^

### Expanding Applications and Ergonomic Interventions

It is important that sensors that have not been utilized to their full potential for work-related activities can be used to expand their application.^173^ For example, the optical fiber sensor, which has been solely utilized to focus on the assessment of the spine in postural stability, can be used to evaluate other risk assessments in the upper limbs or the knees. Wearable sensors can assist in reconfiguring workstations or enhancing them by implementing microbreaks and ergonomic interventions through a better understanding of the work conditions leading to musculoskeletal strain.^10,90,107,117^ This initiative could include strategies promoting flexible work-rest schedules and preventing the buildup of work-related fatigue.^168^

Lastly, wearable systems are important tools in advancing the evaluation and prevention of WRMSDs. These devices can provide precise and consistent input in the workplace, demonstrating their role in improving workplace safety and health protocols. With future projections showing promising widespread implementation of these devices in workplace settings, the challenges facing them must be addressed to make this a possible event. Addressing challenges, with (i) limitations in ergonomic frameworks, (ii) minor inconsistencies in effective monitoring because of accuracy under varying conditions, and (iii) cost-effectiveness, will lead the pathway to the validation of wearable systems. The validation and adaptation of wearables in PPE design will effectively provide advanced methods for assessing biomechanical risk and preventing WRMSDs in occupational health.

## Conclusions

The incorporation of wearable sensor technology into occupational health signifies a significant transformation in the prevention and assessment of work-related musculoskeletal disorders (WRMSDs). In contrast to traditional risk assessment methods, such as questionnaires and observational forms, the utilization of wearable sensors for biomechanical risk evaluation offers significant advantages for real-time, noninvasive monitoring. A comprehensive search of recent wearable sensors designed for the assessment and prevention of work-related musculoskeletal disorders was conducted, analyzing both established and emerging sensing mechanisms. The literature search was done across three databases: PubMed, Scopus, and Web of Science, leading to the identification of 176 papers that underwent meticulous analysis. The established wearable devices, such as IMU and EMG, alongside emerging technologies like optoelectronics, soft wearable sensors, and pressure sensors, offer considerable improvements over conventional assessments by enabling real-time, quantitative, and highly reproducible data collection. This review emphasizes the possibilities of integrating wearable systems within PPE. Furthermore, this review delineates methodologies for enhancing the prevention of biomechanical risks through techniques such as sensor fusion, the digitalization of ergonomic frameworks, and implementing active real-time feedback, thereby offering a thorough comprehension of biomechanical risks.

Although several challenges appear to limit the integration of wearable sensors in PPE. These limitations include susceptibility to environmental interference, mechanical deformation during rigorous tasks, user acceptance and privacy issues, the need for standardization and data interpretation, and the application of these devices on sample sizes to substantiate ongoing research. Consequently, the prospective of wearable sensors shows potential across multiple domains, such as miniaturization, creation of adaptable interfaces, validation through large sample sizes, and enhancement of machine-learning-driven feedback systems.

The real-time quantitative data from wearable devices can guide the development of ergonomic interventions and smart PPE to improve worker health and raise awareness among ergonomic supervisors. This work showcases emerging wearable sensors with the practical needs of workers across various industries and promotes the design of standalone systems within a suitable biomechanical framework. Our work extends to various areas of occupational medicine as well as the development and implementation of wearable sensors, paving the way for future research aimed at enhancing established and emerging wearable technologies. Lastly, this study is considered to enlighten on active monitoring of biomechanical risks, thereby potentially decreasing the occurrence of WRMSDs and related expenses. The future of occupational health is contingent upon the effective combination of innovative tools with established ergonomic practices, thereby facilitating the development of more efficient, data-informed strategies for the prevention and management of WRMSDs.

## Vocabulary

Biomechanical Risk Assessment: A method used to evaluate physical stress and strain on the human body during work tasks, often identifying risks for musculoskeletal injuries.
Wearable Sensors: Electronic devices worn on the body that collect data on posture, movement, and physiological conditions to monitor occupational health and ergonomics.
Inertial Measurement Units (IMUs): A type of wearable sensor that combines accelerometers, gyroscopes, and sometimes magnetometers to track body movement and orientation.
Electromyography (EMG): A technique or device used to measure muscle electrical activity, often indicating muscle strain or fatigue during work.
Sensor Fusion: The process of combining data from multiple types of sensors (e.g., IMU + EMG) to improve the accuracy and reliability of biomechanical assessments.
Personal Protective Equipment (PPE): Gear or garments worn by workers to protect against injury; increasingly integrated with sensors for health monitoring and ergonomic evaluation.
Real-time Monitoring: The continuous, immediate collection and analysis of data during work activities, enabling instant feedback and risk alerts.
